# Infection of the Upper Urinary Tract during Pregnancy

**Published:** 1908-06-06

**Authors:** 


### Obstetrics.

### INFECTION OF THE UPPER URINARY TRACT DURING PREGNANCY.

It is a good rule of practice to examine the urine of every pregnant woman at some period considerably short of term. The reasons for this routine are well-known and established on ancient experience. Of recent years, however, a new indication has been furnished by the increasing frequency of infection in the upper urinary tract, as a complication of pregnancy. Greater frequency of incidence was first noticed in France, but there is now evidence of a similar state of affairs in this country, and a good deal of positive knowledge of the condition has been acquired.

Sometimes this infection of the upper urinary tract is virulent; the organisms reach the kidney by the blood-stream, and in fact, both the pathological and clinical pictures are those of a pyæmia. Other cases are simple local infections, either by continuity along the mucosa from the lower tract, or by the lymphatic vessels. The bacillus coli is responsible for most of the latter class and for some of the former, but the common pyogenic organisms from distant and even trivial lesions are sometimes found.

The morbid condition varies in gravity in different cases, from a mere catarrh of the upper part of the ureter and pelvis of the kidney, to the occurrence of multiple abscesses throughout the cortex; the whole organ may be converted into a sac of pus, or, on the other hand, the suppuration may be strictly peri-renal.

The severe cases, in their origin and course, are distinctly to be classed as acute abdominal emergencies, and surgical intervention should be prompt and radical; nephrectomy has so far been the most successful operation, but it is obviously foredoomed to failure in the rare cases of bilateral affection, and, moreover, nephrotomy, or even exposure and drainage of the peri-renal region without incision of the gland, has been followed, though rarely, by recovery.

Obviously, however, these are not the cases likely to be detected by a routine examination of the urine. It is the mild cases, of insidious onset, about the fifth or sixth month, or soon after the enlarging uterus has begun to exert effective pressure on the ureter, that herald themselves only by aching pain in the loins with tenderness just below the last rib, usually on the right side, to which attention may be drawn by the discovery of a little albumen in the urine. When microscopical examination reveals, instead of a few casts, a sprinkling of pus cells, suspicion must be aroused. Under these circumstances, it is well worth while to have a bacteriological examination of a catheter specimen, and the discovery of the colon bacillus will confirm the necessity for very careful observation; it is to be remembered that the presence of this organism does not alter the acid reaction of the urine in the early stages.

Constipation, especially when obstinate, is a definite, predisposing factor, that should be taken into consideration in diagnosis. The kidney in this type of case is not enlarged enough to be palpable, but there is usually a little elevation of the temperature, with a sharp rise occasionally, perhaps, and just enough malaise to suggest a mild attack of influenza, or even of appendicitis.

In later stages, when the urine holds a large quantity of pus, there is generally no difficulty in recognising the nature of the lesion, but it is well to remember that kidneys already damaged, whether by calculus or possibly by tuberculosis, are liable to an exacerbation under the increasing stress of pregnancy.

Unfavourable as is the prognosis in the severe type of the disease, the outlook in the milder pyelonephritis of pregnancy is by no means bad either for the mother or the child. Abortion is rare, although premature confinement is not uncommon, but delivery is almost always the signal for complete cessation of the symptoms. Ruppaner put it boldly, *cessante causa, cessat effectus*, but though this be true, the condition is generally not so menacing or so insusceptible of other treatment, as to demand premature induction of labour.

The indications for treatment are plain; in the first place, the pressure on the ureter at the brim of the pelvis must be relieved if possible; it is generally exerted upon the right ureter, and the left lateral decubitus often suffices, but sometimes only relieves the right kidney at the expense of the left. In such cases, various expedients have been tried on the continent, such as frequently repeated momentary distension of the bladder, but the benefit is problematical. If posture fails to give benefit, it is some comfort to remember that the pressure may be automatically relaxed as the uterus rises still further out of the pelvis.

Then it is of prime importance to cure the constipation, and to this end no effort must be spared. Finally, something may be done to attack the infective agent, by the use of intestinal and urinary antiseptics; drugs, such as boracic acid, salol, urotropine, helmitol, hetraline, and many others have been used with success, but the choice may be left to the experience of the practitioner.

It is important to bear in mind that the mild cases may become severe, and that then, like those which are acute from the outset, they command a serious prognosis.

One final word may be said about specific treatment of the infection; whether the agent be the colon bacillus, or the staphylococcus, or streptococcus, it is amenable to vaccine therapy. The activity of the organism can be restricted or entirely inhibited, and the general effects of the toxæmia minimised or dissipated. When one kidney has been removed for an acute infection, it may almost be said, in the light of present knowledge, that vaccination as a prophylactic measure in favour of the other kidney, is imperative.

